# Pedestrian Flow Identification and Occupancy Prediction for Indoor Areas

**DOI:** 10.3390/s23094301

**Published:** 2023-04-26

**Authors:** Nikolaos Tsiamitros, Tanmaya Mahapatra, Ioannis Passalidis, Kailashnath K, Georgios Pipelidis

**Affiliations:** 1 Ariadne Maps GmbH, Munich, Brecherspitzstraße 8, 81541 Munich, Germany; 2 Department of Computer Science and Information Systems, Birla Institute of Technology and Science, Pilani 333031, India; 3 Institute for Informatics, Technical University of Munich, Boltzmannstraße 3, 85748 Garching, Germany

**Keywords:** indoor positioning, indoor localization, pedestrian flow analysis, ARMA model, Prophet model

## Abstract

Indoor localization is used to locate objects and people within buildings where outdoor tracking tools and technologies cannot provide precise results. This paper aims to improve analytics research, focusing on data collected through indoor localization methods. Smart devices recurrently broadcast automatic connectivity requests. These packets are known as Wi-Fi probe requests and can encapsulate various types of spatiotemporal information from the device carrier. In addition, in this paper, we perform a comparison between the Prophet model and our implementation of the autoregressive moving average (ARMA) model. The Prophet model is an additive model that requires no manual effort and can easily detect and handle outliers or missing data. In contrast, the ARMA model may require more effort and deep statistical analysis but allows the user to tune it and reach a more personalized result. Second, we attempted to understand human behaviour. We used historical data from a live store in Dubai to forecast the use of two different models, which we conclude by comparing. Subsequently, we mapped each probe request to the section of our place of interest where it was captured. Finally, we performed pedestrian flow analysis by identifying the most common paths followed inside our place of interest.

## 1. Introduction

GPS and satellite technologies are used for navigation purposes, but they are not precise regarding multistorey buildings, airports, and other indoor spaces. Existing technologies have successfully solved the problem of outdoor localization, but indoor localization is still a work in progress. Outdoor localization focuses on latitude and longitude, but in indoor localization, we also need to consider the altitude, as indoor localization includes multistorey buildings. Specifying the floor in a building is a difficult task and requires precision and tremendous amounts of effort. For indoor positioning, we use indoor localization methods. The focus of indoor localization is to estimate the crowd’s position accurately without a breach of the privacy of any individual. We use the network of many devices to locate people inside a building. Currently, many technologies such as smartphones, Wi-Fi, Bluetooth antennas and beacons placed at specific distances are used for indoor positioning. Many Wi-Fi-enabled devices are available, and a whole lot of data is gathered from public Wi-Fi. To improve the user experience, Wi-Fi probe requests are repeatedly broadcast. These probe requests consist of the device’s MAC address, as well as the timestamp, latitude, and longitude of the known access point. Therefore, these probe requests contain time and space information of the device.

There are many uses of indoor localization, for example, in fields such as augmented reality and navigation in shopping malls, airports, and parking lots, tourist locations, hotels, and amusement parks, among many others. Indoor localization helps these industries get an idea about customer preferences, and by tracking their movement, they can understand which part of their physical space the customer is more interested in.

Despite the variety of applications, indoor localization has no fixed standards. Although indoor localization has become easier, analytics of the results remains in the initial stages. Therefore, the succinct contributions made by us in our manuscript include:

1. First, a detailed comparison was performed between the Prophet model developed by Facebook and our implementation of the autoregressive moving average (ARMA) model. The Prophet model is an additive model that requires no manual effort and can easily detect and handle outliers or missing data. On the other hand, ARMA is used to forecast time series values using autoregression (correlation between previous values and future values) and the moving average (past forecasting error). The ARMA model may require more effort and deep statistical analysis but allows the user to tune it and reach a more personalized result by providing users with an option to modify the parameters.
2. Second, we attempted to study human behaviour. Therefore, we performed business analytics on the probe requests we captured using the devices in a particular place. The obtained result consisted of raw data and the uncertainty radius of each captured probe request. Our approach provided a detailed pedestrian flow analysis, which helped discover the paths customers followed in the place where we had captured the probe requests.

## 2. Related Works

### 2.1. Spatial Analytics

Wirz et al. [1] introduced mathematical methods for inferring and visualizing real-time information based on the data collected through people’s mobile phones. This method was tested by tracking the location of attendees of the 2011 Lord Mayors Show in London. GPS and Wi-Fi/GSM fingerprinting were used to derive information regarding location. To receive constant updates, a dedicated application was installed on the mobile phones of attendees. Next, the user’s position at time *t* was determined using the most recent updates on a time interval, then calculating his/her heading direction (θ), which is the angle between the user’s most recent location updates. The information was visualized by applying kernel density estimation. The result was a heat map that illustrated information such as crowd movement, crowd pressure, and density distribution. Although this application provided some important security aspects, the authors could only collect data from users who willingly installed their application, reducing the volume of the data.

Sarshar H. and Matwin S. [2] presented a remote localization technique based on Wi-Fi data and registered data. Access points that were operating in the vicinity of five retail stores continuously recorded any received Wi-Fi signal from Wi-Fi-enabled devices. The registered data were collected offline while the user was inside the range of any of the access points and established a connection with a public network. All this was achieved non-intrusively. For the validation of the method, the authors used real data from five volunteers, then performed the K-sample Anderson–Darling test and two-class and one-class classification to assess the quality of the data and the system. In the end, they concluded that their method can confidently achieve a high accuracy score, mainly on a considerably large volume of data, and that it can operate either alone or in collaboration with other positioning methods.

Prasertsung P. and Horanont T [3] attempted to predict the density of people in a real-world environment through the use of already existing Wi-Fi access points. Their goal was to accurately count the number of users inside a coffee shop using filtering techniques. The test was performed on a weekday when a mid-day discount was available. Probe requests were sniffed through their laptop, which had the necessary software installed. In the end, based on the data that they captured, they showed that during the hours of a promotion day, there was an increase in customers in the coffee shop. A clear limitation of their implementation was that they only considered the Wi-Fi access point of the store to filter the devices outside the store, as their method was not able to remove outside noise or outliers.

Di Luzio et al. [4] presented the deanonymization of the provenance of people who were participating in various large-scale events. This was possible by exploiting Wi-Fi probe requests that people’s mobile phones periodically send to connect to a nearby Wi-Fi network. The data were collected from nationwide scenarios (e.g., political party events), international events (e.g., the resignation of the pope), and citywide scenarios (e.g., in the train station of Rome). They focused their analysis on a user’s preferred network list, which stores, among other information, the user’s last known APs. The authors assumed that any recurrent connection to certain APs signifies the area where the user lives. To achieve the goal, the geographical positions of all these APs were used. For high accuracy, Wigle.net, one of the largest databases mapping APs to GPS coordinates, was used. Through their analysis, they demonstrated the great potential of probe request analysis in human-related phenomena, and, more importantly, they were able to accurately perform deanonymization through the probes that they collected, which ended up matching even ground truth information.

### 2.2. Pedestrian Flow Identification

Weppner et al. [5] presented a crowd monitoring method. Stationary scanners with directional antennas were used to track the devices of people that had their Wi-Fi or Bluetooth activated. The data came from an exhibition at the Frankfurt Motor Show, where 31 directional scanners were used for a period of 13 business days. In the end, to evaluate the results, a video ground truth was performed for 7 of these days. There were two accuracy levels based on which the location of a person was determined: either by assigning it to the location of the best-matching sensor or by calculating the coordinates of the user’s device through their algorithm. To estimate the location, two different localization algorithms were used, among which one was based on received signal strength indicator (RSSI) multilateration and the other of which was based on crowd-sourced RSSI fingerprinting. In the evaluation, they argued that around 90% of visitors could be localized. Moreover, they visualized these results on a heat map, where a high crowd density was marked as red, while low density was marked as blue. In subsequent work, they evaluated detailed crowd conditions such as crowd movement and common patterns.

Marini et al. [6] proposed a context-free semantic localization approach, which was able to accurately recognize and then visualize indoor movements. Moreover, they focused on spaces where every room had a very clear role and purpose for the people operating within it. The goal was to be able to easily work with different underlying localization systems. The approach was tested in a hospital, where a Bluetooth low-energy indoor localization system was deployed. The way the system worked was that people inside the hospital such as nurses and doctors carried a RadBeacon Dot, which is a Bluetooth low energy (BLE)-based beacon that periodically broadcasts its signal. Moreover, Android smartphones running a custom-developed application were anchored on the walls of each room and continuously scanned the surrounding area to capture any BLE beacon. Using this data, the authors represented the movement of people as a string of time-encoded characters, which they visualized. They argued that this representation could facilitate the use of various pattern-matching techniques for analysis, which could open new opportunities for research on mobility and flow.

Fukuzaki et al. [7] developed a system that captured Wi-Fi probe requests. Moreover, an authentic anonymous MAC address probe sensor (AMP sensor) was developed, which collected the MAC addresses out of these captured packets and then uploaded them to an analytical server. The goal was to analyze pedestrian flow through the installation of multiple sensors to prevent any future disasters. To ensure anonymity, the proposed system generates a secure hash value (AMAC) from the captured MAC address of the user. In addition, the system performed two important functionalities: particle pedestrian flow analysis and fluid pedestrian flow analysis. On the one hand, the former functionality meant that they were able to infer a person’s trip data based on their computed trajectory, while the latter functionality focused on creating a complete graph by considering the data of each installed sensor. Subsequently, various real-life experiments were conducted to test their system considering a variety of analytics, such as variation of pedestrian flow, the number of resident persons, and their resident time. In conclusion, they argued that while they could analyze the tendency of the pedestrian flow to an extent, future work would be necessary if more accurate results are required.

## 3. Data Collection & Models

### 3.1. Data Collection

We describe the data collection methods used in our study.

#### 3.1.1. The Localization Method

Raspberry Pis were used as beacon devices. The devices had a range of 70 m, and the ideal distance between the beacons was around 10 m to avoid increment in localization uncertainty. For the data collection purposes, a smartphone equipped with a localization method that follows a particle filter approach was used. After data collection, the smartphone uses its unique user identifier to filter out the data that do not have a precise location tagged. Afterward, all the received signals are plotted on a histogram for clustering purposes using the K-nearest neighbor and elbow methods to identify an optimal number of clusters. To strengthen the localization method, the initial localization model was used in a simultaneous localization and mapping (SLAM)-like approach [8]. This helps to improve the precision of data collection [9].

#### 3.1.2. Uncertainty Radius

The smart device localization history and distance from the nearest access point were the most important variables used to compute the uncertainty radius. Two thresholds were set i.e., $\tau_{d}$ and $\tau_{s}$, the former of which is the minimum estimated distance and the latter of which is the maximum acceptable speed (in meters) in the environment. A distance of 0 to 2 m was suitable, as it does not contain many vague received signal strength values. The speed threshold was set according to the localization environment. The depth (δ) to which the localization history had to be checked was considered. Higher depth was undesired, as older predictions lose their relevance over the time in the case of moving objects. A matrix was created to store the calculated uncertainties and check the recorded localization history. The localization history consists of the captured smart devices, which were identified by their MAC addresses. Every localization prediction had a timestamp and location. If any device had a recorded history, the elapsed time was calculated, and the distance from the last prediction was used to calculate the speed (*s*). Uncertainty ($u_{t}$) was calculated as the sum of the distance (*d*) from the last detection and the maximum of all the minimum estimated distances to the nearest access point and the distance threshold. The computed uncertainty was returned and stored in the array only if the speed was within the defined threshold. In case it was more than the defined threshold, the depth of checking the localization history was decreased, and the last element of the array was removed. The process was repeated until the older prediction was within the speed threshold. If no prediction was obtained within the threshold and all the historical predictions had been removed or the maximum depth had been reached, then the last value was treated as an anomaly, and the minimum computed uncertainty from the list was returned [10].

### 3.2. Prophet Model

Developed by Facebook [11], an approach was proposed that uses a configurable model with interpretable parameters. These parameters are adjusted by analysts with knowledge of the domain but without in-depth knowledge of the model. The model is based on the idea of a decomposable time series model [12]. The components of this model are trend, seasonality, and holidays. The following equation combines all these components:

$$y\left(t\right)=g\left(t\right)+s\left(t\right)+h\left(t\right)+\epsilon_{t}$$

where g(t) denotes the trend function, which models non-periodic changes in the value of the time series; s(t) denotes periodic changes, i.e., seasonality; and h(t) denotes irregular period changes, i.e., holidays. The error term $\epsilon_{t}$ represents any individual changes [11].

This model presents some practical advantages as compared to the ARMA model. First, it can accommodate seasonality with ease and for multiple periods. Second, this model can handle missing data. Third, the parameters of this model can be easily interpreted and adjusted.

#### 3.2.1. Trend Model

The saturating growth model and the piecewise linear model are the two models that are implemented for Facebook applications.The growth model shows how the population has grown in past and how it is expected to continue growing. It follows a non-linear graph that saturates at a certain capacity. The logistic growth model is used to model the growth model. The most basic form of the logistic growth model is:

$$g\left(t\right)=\frac{C}{1+\epsilon^{-k(t-m)}}$$

where *C* represents the carrying capacity, *k* is the growth rate, and m is the offset parameter.

This equation does not capture two important aspects. The carrying capacity is not constant, so it should be replaced with C(t). The growth rate also needs to be changed, as it can be altered by new products.

To embody these changes, some change points are introduced when the growth rate is allowed to change. First, an assumption is made regarding change points. At time $s_{j}$, when j=1,⋯, *S*, there are *S* change points. A vector of rate adjustments is denoted by $\delta \epsilon R^{S}$, where $\delta_{j}$ is the change in rate occurring at time $s_{j}$. The growth rate now becomes $k+\Sigma_{j:t>s_{j}}\delta_{j}$, which incorporates the base rate (*k*) at any time (*t*), along with all adjustments until that point [11]. It can be more cleanly represented as:

$$a_{j}\left(t\right)=\left\{\begin{array}{cc} 1 & if\;\;t\geq s_{j}\\ 0 & 0.3\;\;otherwise \end{array}\right.$$

Therefore, the rate at time *t* is:

$$k+a{\left(t\right)}^{T}\delta$$

The trend model can be more precisely written as:

$$g\left(t\right)=\left(k+a{\left(t\right)}^{T}\delta \right)t+\left(m+a{\left(t\right)}^{T}\gamma \right)$$

#### 3.2.2. Seasonality

The human behaviors represented by business time series are often subject to multiperiod seasonality. For example, school breaks or vacation can produce the effects that repeat yearly. Similarly, a week of 5 working days can produce effects that repeat weekly. To model these periodic effects, Fourier series [13] are used. For *P* representing the regular period expected by the time series, the smooth seasonal effects can be approximated as:

$$s\left(t\right)=\Sigma_{n=1}^{N}\left(a_{n}\cos\left(\frac{2\pi nt}{P}\right)+b_{n}\sin\left(\frac{2\pi nt}{P}\right)\right)$$

For yearly and weekly seasonality, *N* = 10 and *N* = 3 work well for the majority of problems [11].

#### 3.2.3. Holidays and Events

This model includes one more aspect, i.e., holidays, which are difficult to model using a smooth cycle, as they don’t follow any definite pattern. Along with global holidays, the model also provides the ability to construct a custom list according to the country. The union of all these lists is given to the forecasting problem. The effects of holidays are assumed to be independent. For holiday *i*, let $D_{i}$ be the set of past and future dates for that holiday. An indicator is added to identify whether a time (*t*) is during holiday *i*, and each holiday is assigned a parameter ($K_{i}$) representing the corresponding change in the forecast. The days around holidays are treated as holidays as well. Additional parameters are included to account for these days [11].

#### 3.2.4. Modeling

The model permits analysts to incorporate their own knowledge and improve the model without understanding all the underlying statistics.

1. Capacities: The best possible capacities are specified by external data for the total market size;
2. Change points: Knowledge about certain product changes is used to specify change-point dates;
3. Holidays and seasonality: Knowledge of which holidays have the greatest impact on the growth rate in which regions is used to prepare the mode;.
4. Smoothing parameters: A variety of smoothing models enable selection between more global or locally smooth models.

In addition to these factors, the seasonality and holiday parameters provide the opportunity to specify how much of the historical seasonal variation can be expected in the future. Model fitting is further improved by modifying these parameters. The change points are identified from the historical plotted data points, which also provides an overview of factors such as growth, seasonality, and outliers.

### 3.3. The ARMA Model

In this model, the output variable depends linearly on its previous values and on a stochastic term. Time series must be stationary to perform ARMA modeling. For a non-stationary sequence, smoothing is performed before continuing with the forecasting [14]. The mathematical expressions of the autoregressive AR(p) model are described as follows:

#### 3.3.1. Autoregressive Model

$y_{t}=c+\varphi {}_{1}y_{t-1}+\ldots +\varphi_{p}y_{t-p}+\epsilon_{t}$, where $y_{t}$ is the stationary time series, *c* is a constant, *p* is the order of the AR model, $\varphi_{j}\left(j=1,2,\cdots ,p\right)$ are the parameters of the autoregressive model, and $\epsilon_{t}$ (white noise) is the sequence of the distributed independent random variables.At the same time, $E(\epsilon_{t})=0$, $Var\left(\epsilon_{t}\right)=\sigma_{\epsilon }^{2}>0$ is also satisfied.

#### 3.3.2. Moving Average Model

In this model, the future values are predicted by the linear combination of past and present values. The mathematical expression of the moving average model MA(q) is described as follows:

$$y_{t}=E_{t}+\theta_{1}\epsilon_{t-1}+\cdots +\theta_{q}\epsilon_{t-q}$$

where $y_{t}$ is the stationary time series, *q* is the order of the MA model, $\theta_{j}\left(j=1,2,\cdots ,q\right)$ are the parameters of the model, and $\epsilon_{l}$ (white noise error term) is the sequence of the distributed independent random variables. At the same time, $E\left(\epsilon_{l}\right)=0,Var\left(\epsilon_{l}\right)=\sigma_{\epsilon }^{2}>0$ is also satisfied.

#### 3.3.3. Autoregressive Moving Average

The autoregressive moving average is the combination of the autoregressive model and the moving average model. The mathematical expression of this stationary random process is described as follows:

$$y_{t}=\varphi_{1}y_{t-1}+\cdots +\varphi_{p}y_{t-p}+c\epsilon_{t}+\theta_{1}\epsilon_{t-1}+\cdots +\theta_{q}\epsilon_{t-q}$$

where $\varphi_{1},\varphi_{2},\cdots ,\varphi_{p}$ are the autoregressive model parameters, *p* is the order of the autoregressive model, $\theta_{1},\theta_{2},\cdots ,\theta_{q}$ are the moving average model parameters, *q* is the order of the moving average model, *c* is a constant, and $\epsilon_{t}$ is a white noise sequence with $y_{t}$ distributed independently and identically. At the same time, $E\left(\epsilon_{t}\right)=0,Var\left(\epsilon_{t}\right)=\sigma_{\epsilon }^{2}>0$ is also satisfied. If p=q=0, the model is reduced to $y_{t}=\epsilon_{t}+c$, which is a white noise sequence.

#### 3.3.4. ARMA Modeling Procedure

The general application process is [15,16]:

1. To determine the order of the ARMA model, a three-step method is used. In the first step, the autocorrelation function and partial differential coefficients are calculated. Secondly, the Akaike information criterion and the Bayesian information criterion (BIC) of various models with different values of p and q are compared. Model with lower criterion values perform better. Lastly, to fix the model order, the final prediction error (FPE) is used;
2. Parameter estimation was performed using a variety of methods including least squares estimation, moment estimation, and direct estimation of the autocorrelation function;
3. The randomness is verified by testing the residual sequence of the model using white noise;
4. Continued testing is performed to establish an estimation model until an optimal model is obtained;
5. The forecasting model is then applied to the optimal model.

#### 3.3.5. Evaluation Metrics

The model was evaluated using a statistical comparison of model estimates or predictions $\left(P_{i};i=1,2,\cdots ,n\right)$ against reliable and pairwise matched observations $\left(O_{i};i=1,2,\cdots ,n\right)$. The average model estimation error is written as:

$${\bar{e}}_{\gamma }={\left[\frac{\Sigma_{i=1}^{n}w_{i}{\left|e_{i}\right|}^{\gamma }}{\Sigma_{i=1}^{n}w_{i}}\right]}^{\frac{1}{\gamma }}$$

where γ≥1, and $w_{i}$ is a scaling assigned to each $|e_{i}{|}^{\gamma }$ according to its influence on the total error.

Mean absolute error, root mean squared error, and R squared (coefficient of determination) are used as evaluation metrics.

## 4. Conceptual Approach

### 4.1. System Architecture

**Figure 1:**
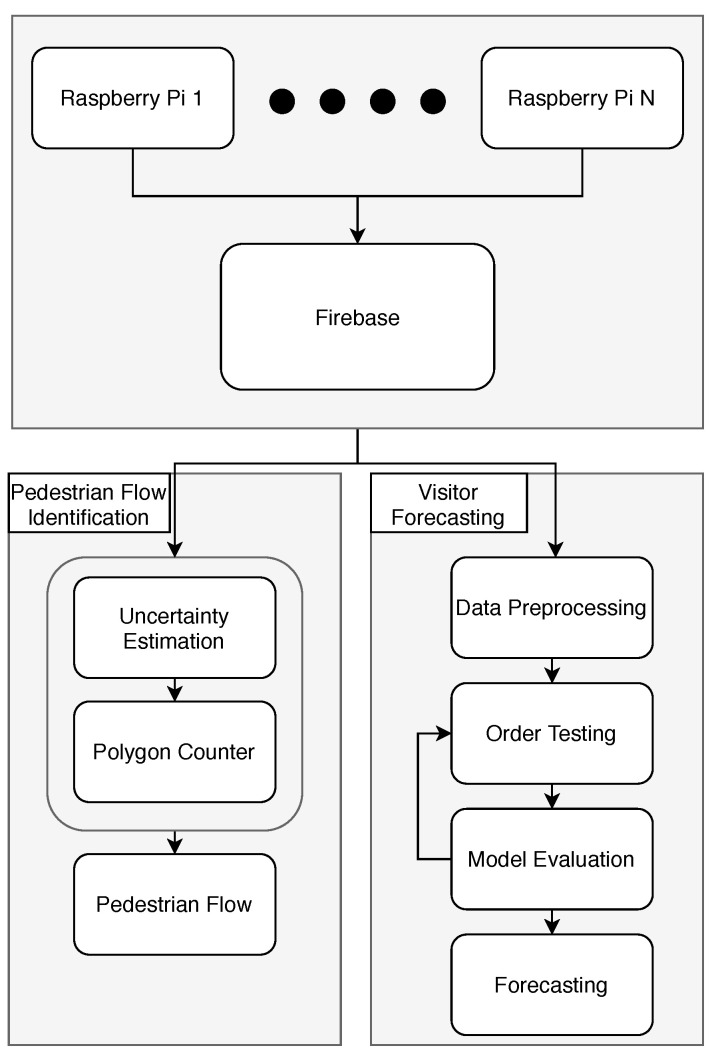
Architecture of the system.

The main components of the architecture (Figure 1) include:

**The data collection and storage component** comprises the necessary hardware for the purpose of data collection and a database used to store real-time data.

1. Raspberry Pi machines are responsible for capturing the Wi-Fi probe requests from customers’ smart devices;
2. Firebase is used to store the real-time data.

**The analysis component** is performed in Python and incorporates two distinct paths.

**Polygon counter and pedestrian flow**

1. The path maps the captured probe requests to its corresponding machine by evaluating the possible uncertainty radius;
2. The obtained result is further used to identify the most common paths that the customer followed inside the place of interest.

**Visitor Forecasting**

1. The captured are analyzed and manipulated to obtain the data in the required form without any missing values or any other possible discrepancy;
2. The model is tested for various orders until the best model is obtained;
3. After obtaining the model, the forecasting model is applied.

### 4.2. Data Description

A mall in Dubai was chosen for data collection. The collected data were uploaded to Firebase, a real-time database in which all the captured data ae stored. The data stored in Firebase include the hashed MAC address of the user, the latitude and longitude of his position, uncertainty radius, and the timestamp of the captured probe request. The Hashed MAC address distinguishes every unique user. The uncertainty radius represents how far away the captured probe request was from the individual machine. The uncertainty radius is important in polygon counter analysis, in which the possible uncertainty of each captured request is considered. Another important aspect of the analysis is the geoJSON file, which consists of the exact coordinates of each polygon inside the place of interest. The captured information is used to map each probe request to the location where the probe was captured. Table 1 depicts a sample dataset for polygon and pedestrian flow analysis.

### 4.3. Forecasting

**Figure 2:**
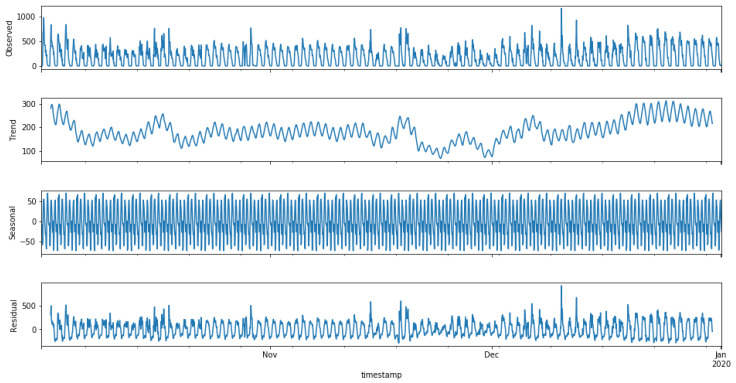
Decomposition of data.

The collected data had a series of discrepancies, which were removed before forecasting, as they can skew the model and produce unrealistic forecasts. The analysis was performed on an hourly basis; therefore, the time zone and daylight saving were taken into account. As the collected data span from October 2019 to December 2019, daylight saving was considered. To fill in missing or incorrect values (ARMA needs continuous data for better forecasting), a list for each day of the week was made, and any missing values were filled in with the mean of all other valid values of that specific day. This ensured minimal loss of information. Loss of information is a tradeoff for better performance of the ARMA model; this tradeoff has a minimal impact on forecasting since artificial values are not created arbitrarily but existing values are used to find the most suitable value. The modified data are statistically decomposed, i.e., deconstruction of the data in the trend using seasonal and residual components. Figure 2 shows an illustration of all the components. Every peak in the graph shows the hour of a particular day with the highest number of probes captured. The data trend is dependent on the number of probes captured on the next day. The trend is upward if more probes were captured on the next day and vice versa. The seasonality of the data stayed the same for the whole period of three months. Variation in seasonality is expected over an extended period only. Spikes at random intervals in the residual graph were considered noise.

**Figure 3:**
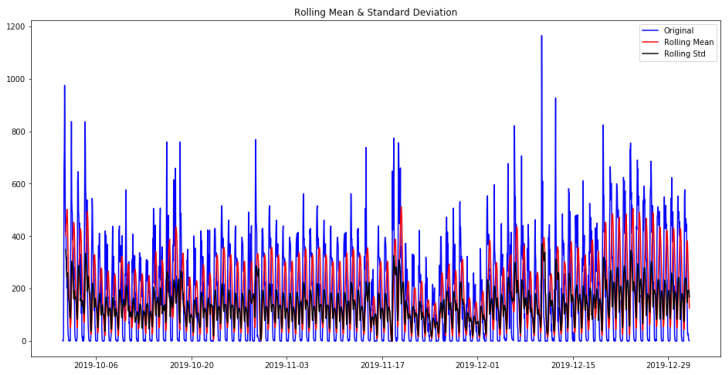
Verification of the stationarity of our data.

Two methods were used to checking the stationarity of the time series:

1. In the first method, the data were visualized with two rolling statistics. In Figure 3, blue represents the original data, while red and black indicate the rolling mean and the rolling standard deviation, respectively. For the time series to be stationary, both the rolling statistics should remain constant over time. Figure 3 shows that the rolling statistics are parallel to the x- axis, and the varying upward and downward movements are due to the varying number of probes observed within a day.
2. In the second method, the Dickey–Fuller test was used for more accurate assessment. The null hypothesis (i.e., no statistical relationship is present between two sets of observed data and measured phenomena for a given variable) was that the time series is non-stationary. By running the Dickey–Fuller test, we obtained the following results: (i) ADF statistic: −4.494521444852915, (ii) *p*-value: 0.00020117099690995828, (iii) Critical Values: 1%:−3.4333504627066542; 5%: −2.8628655035890977; 10%: −2.567475631233297

With a *p*-value this small with an ADF statistic smaller than the critical values at 1%, 5%, and 10%, the null hypothesis was rejected.

**Figure 4:**
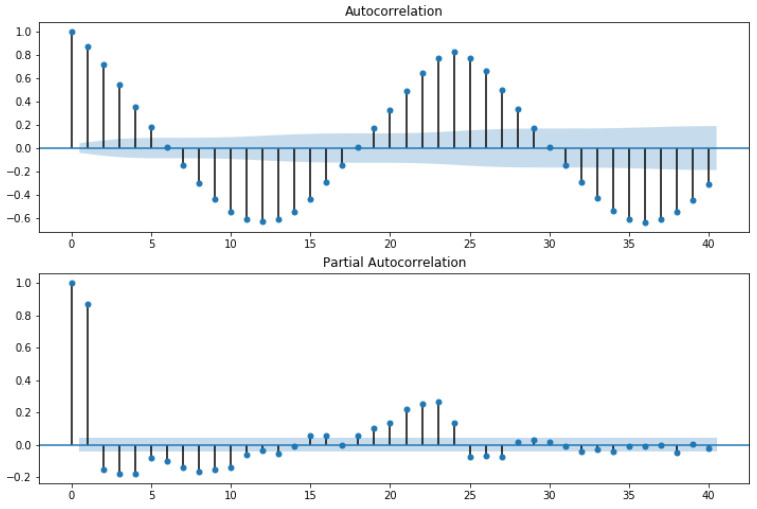
Autocorrelation and partial autocorrelation.

The stationarity of the data was checked using the Dickey–Fuller test. After ensuring that the data were stationary, the order for the ARMA model was determined. Two distinct approaches were followed:

In the first approach, autocorrelation function (ACF) and partial autocorrelation function (PACF) graphs were used. The ACF denotes the correlation between the series and the past values, whereas the PACF denotes the correlation between the present value and past value without considering any intermediate values. ACF was used to determine the optimal number of MA terms, and PACF was used to determine the optimal number of AR terms. ACF precisely shows the correlation between the observation at a current point in time and the observations at all previous points in time. On the other hand, PACF, as a subset of ACF, shows the correlation between observations made at two points in time while accounting for any influence from other data points. The chosen AR and MA terms also determine the order of the model.To choose the correct terms that should be used in the model, Figure 4 was considered. The considered MA term was 6, and the AR term was 2.

The horizontal blue line represents the significant thresholds, whereas the vertical lines represent the ACF and PACF values at a certain point in time. Vertical lines that exceed the horizontal lines were considered significant. As shown in Figure 4, there are six lines that exceed the blue horizontal lines before the first point inside the blue lines. Therefore, the MA term considered was 6. According to the same principle, in the lower plot of Figure 4, there are two vertical lines that are above the horizontal blue line before a vertical line that passes under the significant bound for the first; therefore, the AR order was fixed as 2.

**Figure 5:**
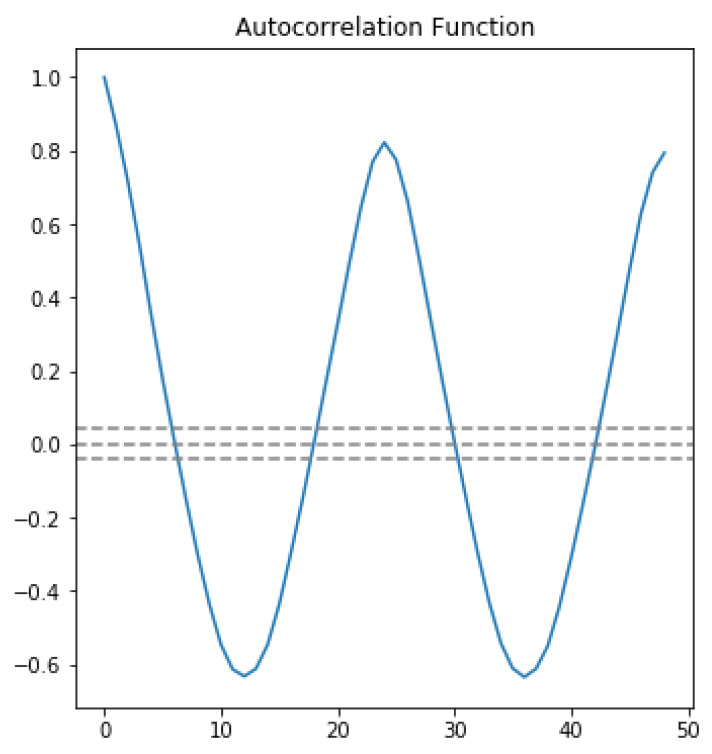
Autocorrelation line plot.

**Figure 6:**
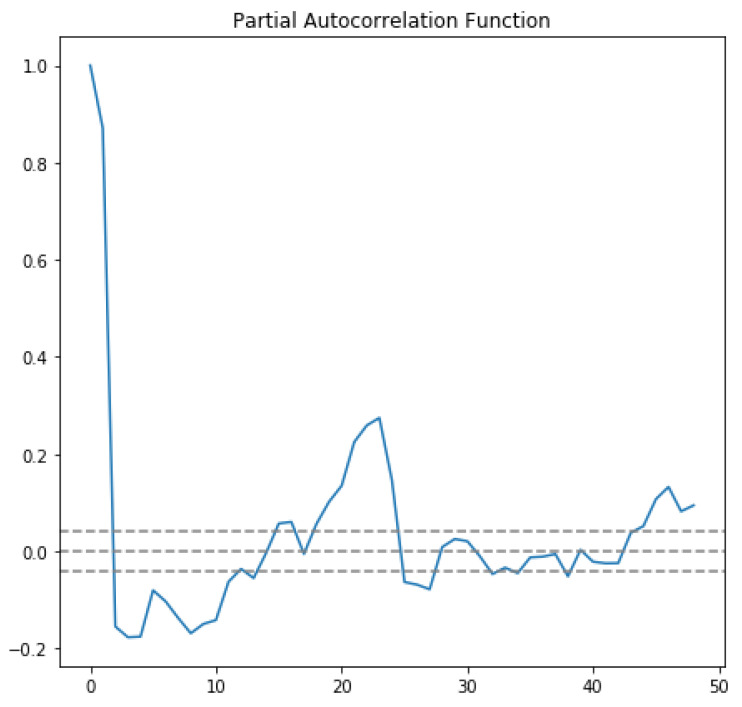
Partial autocorrelation line plot.

In order to feel certain about our choice, we plotted ACF and PACF as line plots. Figure 5 shows the line plot of ACF, and Figure 6 shows the line plot of PACF. In both plots, the three horizontal dashed lines indicated the significance bound, and our line plot is only considered significant when it reaches the upper bound for the first time. As shown in Figure 5, the line reaches the upper bound for first time when the value is 6, assuring that the value of the MA term is 6. Similarly, in Figure 6, the line descends and reaches the upper bound when the value is 2, assuring that the value of the AR term is 2.

In the second approach, the model was fit 12 times with 12 different combinations of AR and MA terms. Then, every model was examined with the AIC score, BIC score, and fit time. The AIC (Akaike information criterion) and BIC (Bayesian information criterion) indicate how the well the data fit the model. The lower the AIC and BIC values compared to other models, the better the fit is. As shown in Table 2, the order (2, 0, 6) has the lowest AIC and BIC scores, as well as a considerably short fit time. This result confirms that the best order to use for our model is (2, 0, 6).

### 4.4. Polygon Counter

**Figure 7:**
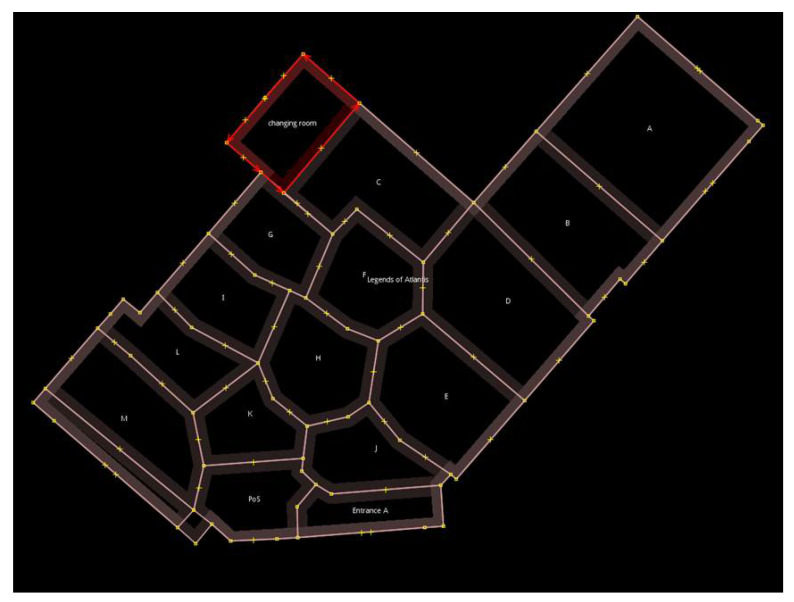
Polygons of the store.

The next task involved the mapping of probes to the section where they were captured. The main motive for doing so was to determine which section gathers the most probe requests. The store was divided into a number of polygons, and each polygon represented a different section of the store. Figure 7 shows the layout, along with the polygons for the store, which were taken into account. The areas with products were named using letters A to M, and other areas such as entrances, changing rooms, and the cash counter were represented as Entrance A, Entrance B, Changing Room, and PoS, respectively.

**Figure 8:**
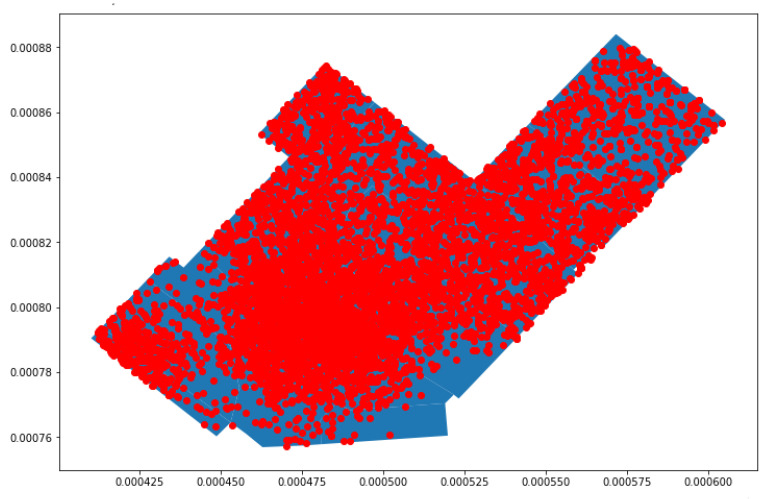
Mapping of probe requests for a common day.

Analysis was performed on a common day by plotting each probe request captured in the store. Figure 8 shows the probe requests distributed in various polygons. It was observed that many probe requests are concentrated in the center, along with a significant number in other sections. The first observation was that customers prefer entering the store from Entrance B instead of Entrance A.

The aim was to find the polygon where the customers showed the most interest. Two different approaches were followed, then evaluated and compared. The approaches were as follows:

- In the first approach, every probe request was mapped to the polygon where it was captured, increasing the polygon count by one;
- In the second approach, only probe requests within a small uncertainty radius were considered. In this approach, the polygon count was increased when a probe request was captured within a given polygon an when the uncertainty radius of a different probe request intersected. The uncertainty radius of each probe request was checked, and the probes with a high uncertainty radius were removed, as such probes led to inaccurate results.

**Figure 9:**
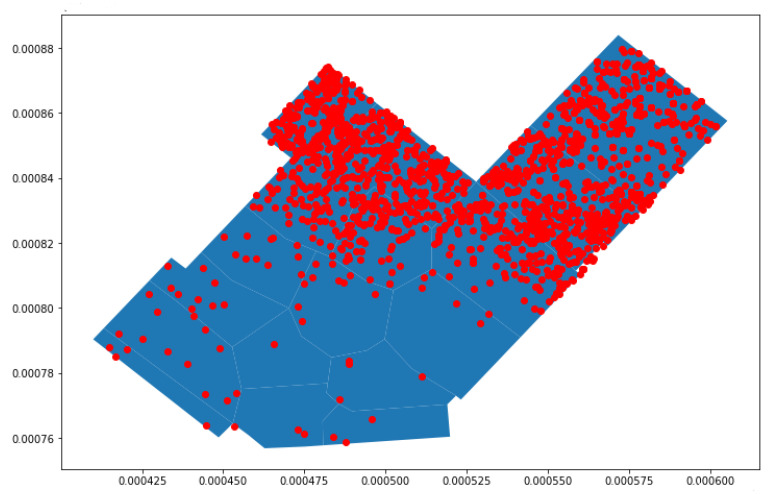
Proberequests with an uncertainty radius greater than 5 m.

As shown by the plot in Figure 9, for the probes with an uncertainty radius of more than 5 m, it was observed that many of the probe requests that were captured in polygons A, B, and C and in the changing room were uncertain, as many of the captured probe requests either fell outside the store or in a different polygon of the store. For this reason, the uncertainty radius was reduced to 4 m; then, the probe requests were mapped.

**Figure 10:**
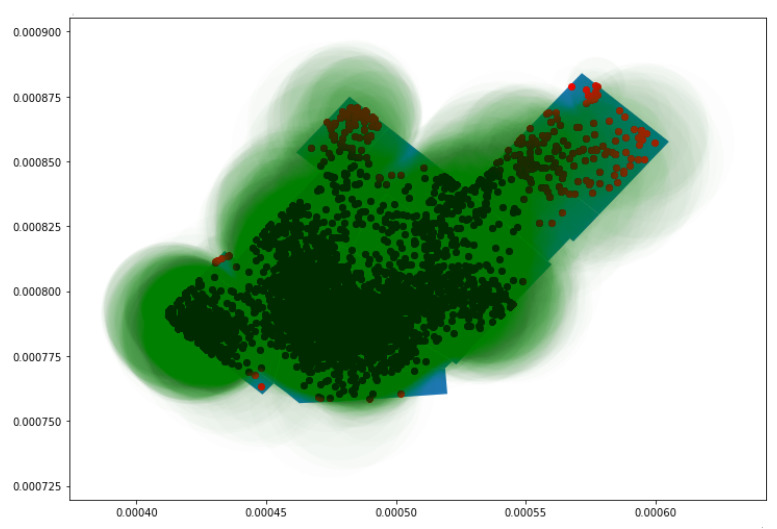
Probe requests with an uncertainty radius less than 4 m with their uncertainty circles.

Figure 10 illustrates the second approach in more detail, in which probe requests captured with an uncertainty radius of less than 4 m are plotted. It was observed that majority of probe requests were captured around the center of the store and the surrounding polygons. Around each of the probe requests, a green uncertainty circle was created, and its size was based upon the radius of each probe request. The polygon count was increased not only by considering the position of each point but also based on when the uncertainty circle of a point outside of the polygon intersects with the polygon of concern. This implies that every probe, depending on the size of its circle, might be considered for multiple polygons.

### 4.5. Pedestrian Flow

The last task was to identify the most common paths that customers take while inside the store. Initially, the path that each customer followed was determined; then, the same paths were summed to obtained the result. To accomplish this task, every polygon in which each unique probe was captured was determined. It was observed that as the store is not very large, the majority of the captured probes were in only one or two polygons. The main reason for this was the time gap between Wi-Fi probe requests. Usually, one probe request was followed by another after 5 to 10 min, and during this interval, most of the customers had already finished their business and were never captured again. There were rare exceptions of the capture of an individual more than once. Most such instances were employees of the store, with multiple captures during the day. These exceptions were not taken into consideration, and the focus was on customers that were captured twice.

Every month was examined separately because comparison was desired between two months, in addition to providing important information such as the common patterns repeated or changed in the following month. Another reason for separate examination was related to the data available for each month. For some days in the months of October and November, no data were received due to faulty machines. Therefore, there were fewer common patterns for these months compared to December.

## 5. Results

In this section, the observations of pedestrian flow analysis are presented. Fewer data were available for the months of October and November; therefore, fewer common paths were observed compared to the month of December. Moreover, in the month of November, despite having more faulty or missing dates, the same number of paths was produced as for the month of October but with fewer occurrences. Owing to the fewer occurrences, there was a lack of confidence to back up these results; therefore, the validity was checked by comparing the results with the common paths observed in other months.

As discussed earlier, the most common paths were the addition of the paths of customers who were captured in two polygons. The results for the months of October and November are shown in Table 3 and Table 4 respectively.

**Figure 11:**
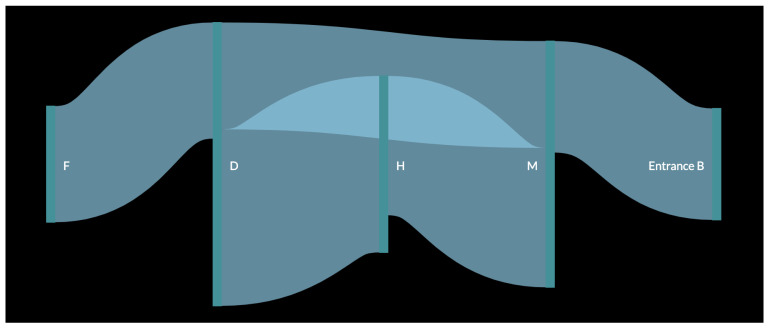
Customer flow for the month of October.

**Figure 12:**
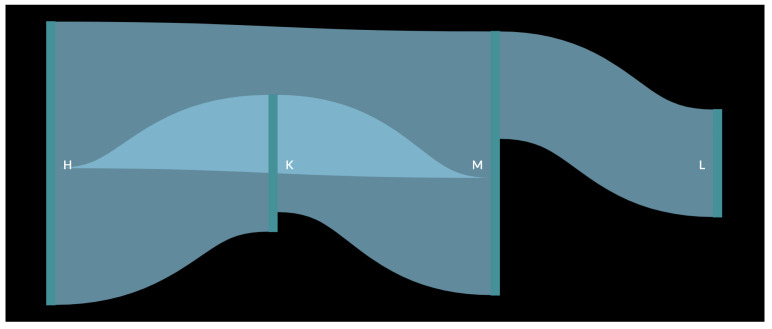
Customer flow for the month of November.

In order to better understand the result, a pedestrian flow of the data was created (Figure 11 and Figure 12). In the figure, the data for each month were used as input for the creation of one large pedestrian flow consisting of all the common paths found in the original analysis. This resulted in the creation of a more complete path that a customer would have followed while inside the store. It was observed that as in the figure with a smaller path from one vertical green line to another, all these connected to form the whole pedestrian flow. The thickness of the flow from one polygon to another depends on the number of small paths recorded. For example, Figure 11 shows that path DH has the thickest flow, as it was captured 38 times, which is more than any other path for the month of October. Similarly, in Figure 12, the thickest flow is for HM, although its thickness is smaller in scale, as it does not differ considerably from other paths.

**Figure 13:**
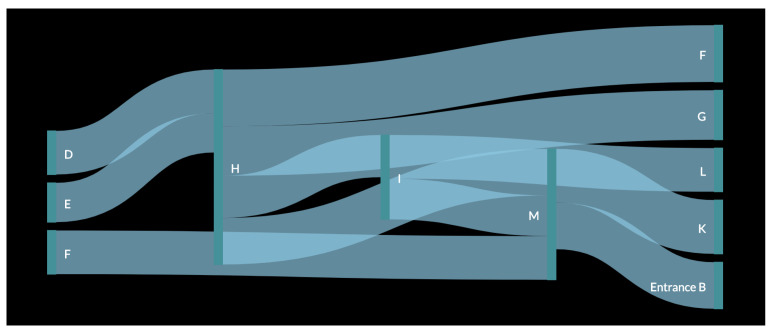
Customer flow for the month of December.

The first observation was that there is a repeated occurrence of polygons H and M, which can be explained by the fact that M is near Entrance B and, as it was observed that customers prefer Entrance B over Entrance A, they are bound to pass through this polygon as well. It was also inferred that H, which is in the middle of the store, was also visited by several customers. As observed in Figure 11, after passing these two initial polygons, customers moved towards polygons D and F. This created a common path for the month of October, i.e., Entrance BMHDF. Similarly, for the month of November, the final common path was LMKH (or MLKH), as observed in Figure 12. For the month of December (Figure 13), more paths were observed, as more data were collected for every day of the month. The results for this month are shown in Table 5.

Repeated occurrence of important polygons H and M was observed, while other polygons, such as E and I, were captured for the first time as part of the path. The occurrence of the new polygons could be the result of either having more data or of greater interest shown by customers in these sections in comparison to previous months. Furthermore, more final common paths are those which a customer could have followed while moving through the left side of the store. The final path is LIGF.

Finally, it was concluded that majority of the customers preferred to enter through Entrance B and move around the central and left side sections of the store. The most common final paths after the three-month analysis are as follows:

- Entrance BMIHD;
- MKHF;
- MLKH;
- LIGF;
- MHED.

In conclusion, we proved that the most common paths that the customers preferred while inside the store can be constructed. The customers considered in the analysis were those customers who were captured twice during their time inside the store. Moreover, the results seem to be in line with the polygon counter analysis, which is presented in the following section.

## 6. Discussion

In this section, the forecasting results are presented, and the two models used for forecasting are compared.

### 6.1. Forecasting

**Figure 14:**
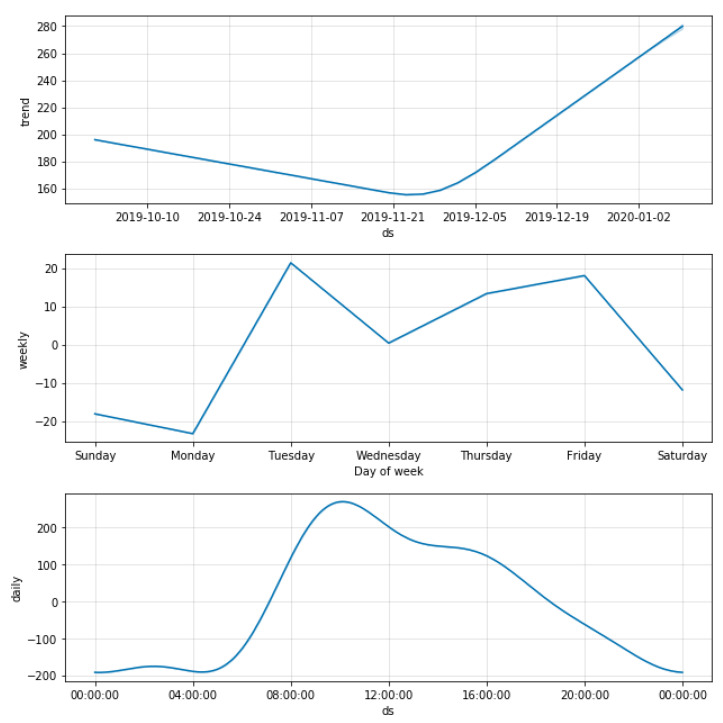
Data components using the Prophet model.

Figure 14 shows results that were observed by implementing the Prophet model. Valuable results were obtained with respect to analytics such as underlying trends of the data in both weekly and daily duration. The first observation was that the trend of the data had a downward movement initially but started trending upwards as December approached. This may have occurred for various reasons, the most important being the Christmas period. It was clear from the daily trend that the store was most crowded at 9 A.M., which is also its opening time. This makes sense, as the store is part of the hotel, and most people might prefer to shop early in the morning before the start of their day. The trend then falls steadily until the store’s closing time at 8 P.M.

**Figure 15:**
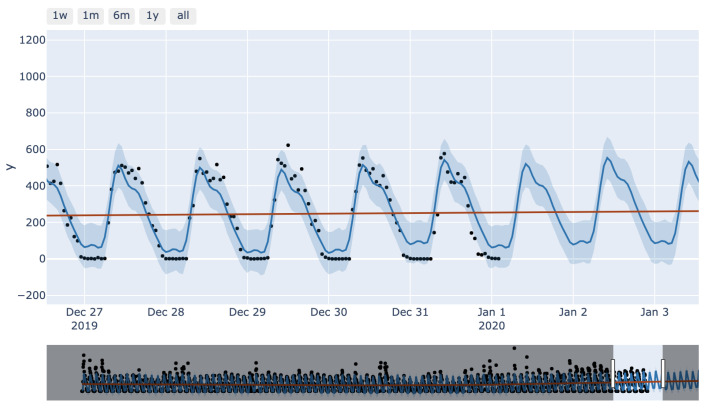
Prophet interactive plot.

Another advantage of Prophet implementation is how easy it was to plot interactive graphs such as that shown in Figure 15. In this graph, a forecast can be achieved by specifying the data or part of the data in two distinct places. In the upper left corner, data, weekly, monthly, yearly, or all of the above can be observed, while the lower bar specifies which part of the data is of interest. All these features would be of great help when a particular time period of data is inspected.

**Figure 16:**
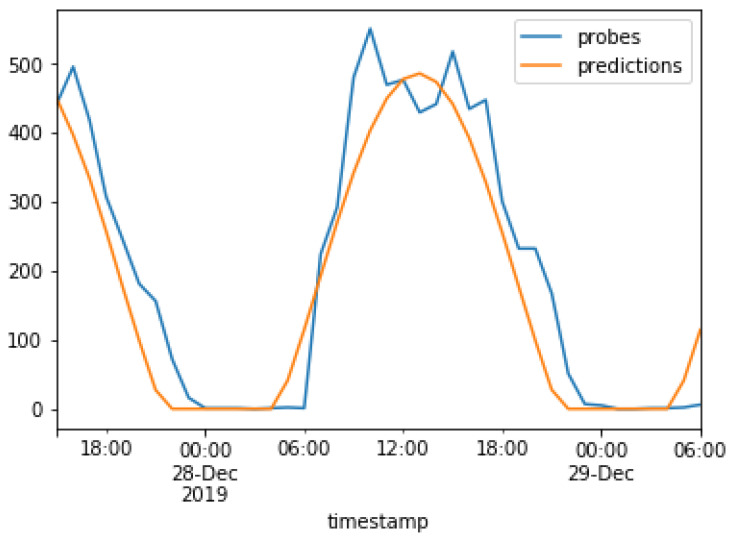
Predictions of the ARMA model.

**Figure 17:**
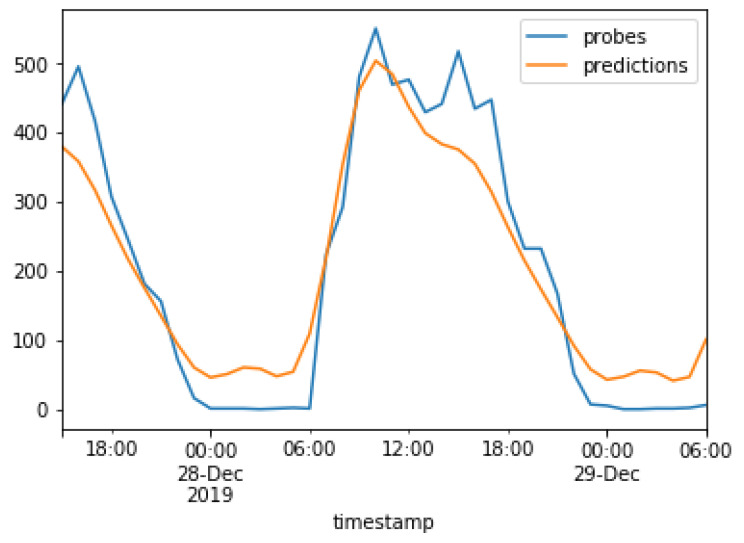
Predictions of the Prophet model.

The results of the models used in the present study are compared next. The real values are plotted against the predicted values for the ARMA model and the Prophet model in Figure 16 and Figure 17, respectively. The real values, i.e., captured probe requests from beacons, are indicated in blue, while the predictions are indicated in orange. This graph was plotted after the training period of 3 months which, as discussed earlier. This graph was plotted for a duration of 40 h, from 6 P.M. on 27 December to 6 A.M. on 29 December.

Figure 16 shows that the ARMA model follows a sigmoid curve and was able to predict the hours when no probe requests were captured, while the Prophet model attempts to capture spikes in the original data.

Table 6 and Table 7 show the performance of the approaches based upon evaluation metrics MAE, MSE, RMSE, and R${}^{2}$. The two approaches are close to each other in terms of all evaluation metrics. The values suggest that the approaches fit well with the data and that the predictions are also quite accurate. The data also show that the Prophet model was quite accurate and easy to implement but that similar results can be obtained by performing statistical analysis using the ARMA model. Although the ARMA model was not easy to use, it provides an opportunity to tackle complex cases by deepening the analysis, which was not possible using the Prophet model.

### 6.2. Polygon Counter

**Figure 18:**
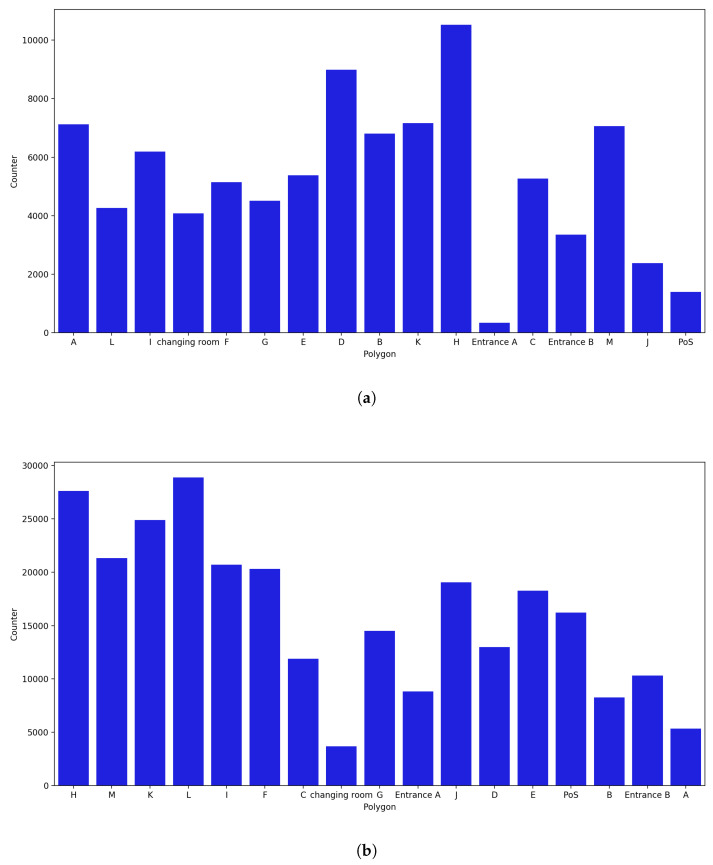
Polygon counter for the month of October. (**a**) Polygon counter without considering the uncertainty. (**b**) Polygon counter considering the uncertainty radius.

Polygon counter analysis was implemented separately for the months of October, November, and December, with each month showing distinct features. Even with a small number of probe requests, the majority of the polygons have an increased counter, as shown in Figure 18b as compared to the polygons in Figure 18a. This was a result of considering the uncertainty radius. The polygons with the fewest probes are those that captured mostly uncertain probe requests.

**October Counter:**

As shown in Figure 18a, the normal approach was used, in which the counter of a polygon is increased only when a probe request is captured within the polygon. On the other hand, as shown in Figure 18b, probe requests with an uncertainty radius smaller than 4 m were also considered. When uncertainty was not considered, it was observed that H was the polygon with the most captured probe requests and with a clear gap relative to the polygon with the second most captured probe requests, i.e., polygon D. This was validated by the fact that H was the polygon with most occurrences in the most common path, as discussed earlier. On the other hand, Entrance A and Entrance B, along with the PoS, changing room, and polygon J, are the polygons with the fewest captured probe requests. As previously observed, Entrance A was not preferred by most of the customers, and Entrance B is the first polygon that customers pass through as they enter the store.

As shown in Figure 18b, H remains one of the polygons with the most captured probe requests, whereas all other polygons have changed. This is validated by the fact that during the analysis for the month of October, polygons A, B, and D had some captured requests with an uncertainty radius of more than 7 m. Thus, when an uncertainty radius of less than 4 m is considered, A and B are among the polygons with the fewest captured probe requests. Furthermore, Entrances A and B and the changing room are the polygons with the fewest captured probes.

**November Counter:**

**Figure 19:**
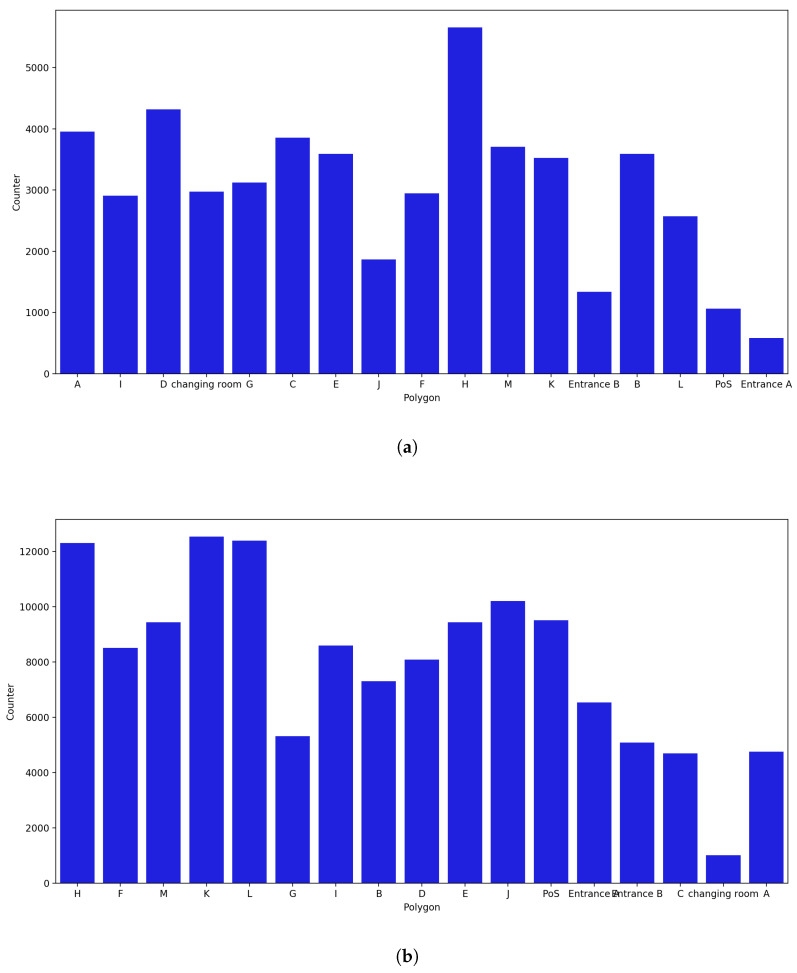
Polygon counter for the month of November. (**a**) Polygon counter without considering the uncertainty. (**b**) Polygon counter considering the uncertainty radius.

The least amount of data was available in November because for the initial 9 days, the beacons were not fully functional. Polygons H, D, A, and M recur in the list of polygons with the highest number of probe requests captured when the uncertainty radius was not considered, and the polygons with the fewest captured probe requests were Entrance A, Entrance B, PoS, and J (Figure 19). When the uncertainty radius was considered, polygons A, C, D, and M were no longer among the polygons with the most captured probes; specifically, A and C were among the polygons with the lowest values. Polygon H was still in the top position, while polygons L and J, and PoS were at the top of the list, despite having had only a small number of captured probe requests in the previous month.

**December Counter:**

**Figure 20:**
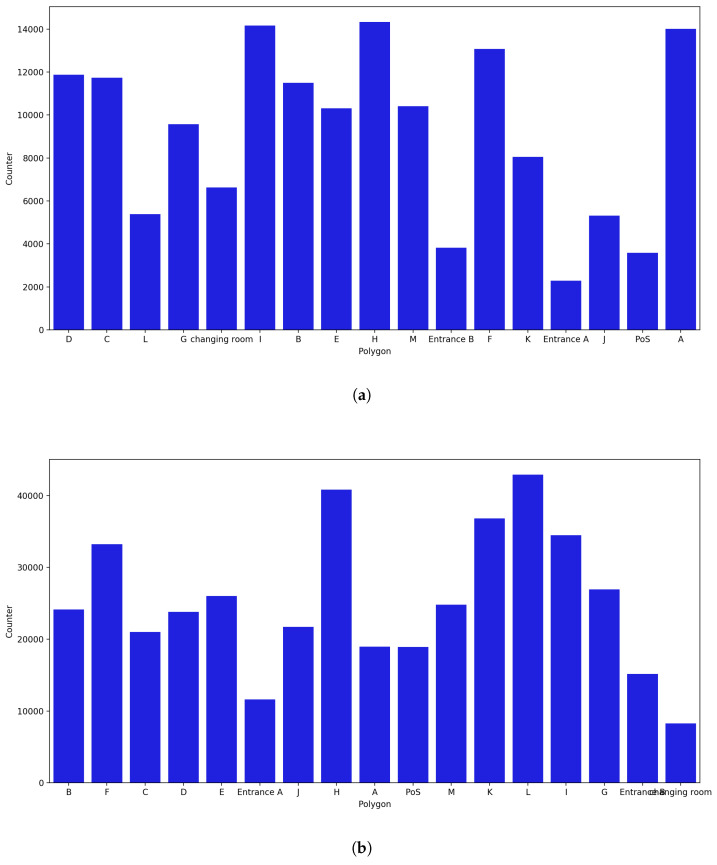
Polygon counter for the month of December. (**a**) Polygon counter without considering the uncertainty. (**b**) Polygon counter considering the uncertainty radius.

Data for all the days were present, resulting in every polygon having the highest number of captured probe requests (Figure 20). Repeated occurrence of polygons A, H, and D in the list of polygons with the most captured probe requests was observed, and new polygons such as F and I were also included in this list. On the other hand, Entrance A, Entrance B, J, and PoS were once again in the list of polygons with the lowest number of captures. Considering the uncertainty radius, it was noticed that H remained in the top places, along with polygon L, but polygon A fell to the bottom of the list.

A different result was obtained from the overall polygon counter analysis when the uncertainty radius was considered. The results were similar for every month under examination. It was also observed that not considering radius was not the ideal approach and could lead to inaccurate and unsafe results.

## 7. Conclusions

We have presented a comparison between two forecasting models and performed polygon counter and pedestrian flow analysis based on spatial data. These data were collected by capturing Wi-Fi probe requests from the smart devices of customers and a variety of spatiotemporal information that is useful for investigating customer behavior. Our data were collected through Raspberry Pi machines installed in the store, and the collected data were stored in real time in an online database.

The first part of the implementation is polygon counter and pedestrian flow analysis. By checking the coordinates of each captured probe request, we mapped each request to the respective polygon where it was captured. However, not considering the uncertainty radius might lead to inaccurate results; therefore, we decided to consider the uncertainty radius for each probe by creating an uncertainty circle around each probe. In this approach, we increased the counter of a polygon not only when a probe request was captured within that polygon but also when the circle intersected any polygon. As a result of polygon counter analysis, we found the pedestrian flow of each customer. Then, a list was prepared with the preferred paths that customers followed based on the most repeated customer paths.

The second part of our implementation was comparing two distinct forecasting models, i.e., ARMA and Prophet. The Prophet model followed a straightforward approach and was easy to implement, whereas the ARMA model required statistical analysis to tune it accordingly. We considered the error in the polygon counter analysis to avoid inaccurate results. The pedestrian analysis resembled a polygon counter, as the polygons with most occurrences were repeated in most common paths. We also observed that despite their different implementations, both the forecasting models could produce accurate predictions. A more extended period of data would be required to check monthly or yearly seasonality.

Due to the unavailability of ground truth, there is uncertainty regarding the validity of our pedestrian flow analysis results. As the relevant store is in Dubai, we could not acquire the ground truth. Additionally, to examine the seasonality, data would need to be collected for the whole year. These points form the basis for our future research. 

## Figures and Tables

**Table 1:** A review of our data for polygon and pedestrian flow analysis.

HashedMAC	Geometry	Radius
000d41df8ad6728f301da..	POINT(55.11857952229, 25.13185977061)	11.223525417245
007763f2e4d7343508163..	POINT(55.11846697934, 25.13179826293)	2.0478777459454
ff78d3800279f4e42bba5..	POINT(55.11854729100, 25.13183920911)	6.7372352781215
ff89ea5817925a291686b..	POINT(55.11856971633, 25.13184948218)	11.223525417245
ff8a383ce79e4d215e595..	POINT(55.11853687957, 25.13181656883)	5.683281756084303

**Table 2:** Testing different orders for the ARMA model.

Order	AIC	BIC	Fit Time
(2, 0, 5)	25,579.120	25,630.422	2.197 s
(0, 0, 0)	29,396.626	29,408.026	0.002 s
(1, 0, 0)	26,266.725	26,283.826	0.046 s
(0, 0, 1)	27,668.724	27,685.825	0.134 s
(1, 0, 5)	26,131.341	26,176.943	0.695 s
(3, 0, 5)	nan	nan	nan seconds
(2, 0, 4)	25,669.154	25,714.757	1.808 s
(2, 0, 6)	25,564.512	25,621.515	2.680 s
(3, 0, 7)	25,566.478	25,634.882	3.281 s
(1, 0, 6)	26,208.346	26,259.649	1.003 s
(3, 0, 6)	nan	nan	nan seconds
(2, 0, 7)	25,565.527	25,628.230	1.630 s

**Table 3:** Preferred paths for the month of October.

Path	Count
DH	38
HM	30
FD	25
MEntrance B	24
DM	23

**Table 4:** Preferred paths for the month of November.

Path	Count
HM	15
HK	14
KM	12
ML	11

**Table 5:** Preferred paths for the month of December.

Path	Count
HF	39
MK	37
HG	34
HM	32
MEntrance B	32
FM	30
IL	30
DH	30
HI	29
IM	28
EH	27

**Table 6:** Evaluation metrics for the ARMA model.

Evaluation Metric	Value
MAE	50.87070012059289
MSE	4843.434247281739
RMSE	69.59478606391242
R${}^{2}$	0.8763698350227241

**Table 7:** Evaluation metrics for the Prophet model.

Evaluation Metric	Value
MAE	53.023252192213384
MSE	3880.3035389025126
RMSE	62.292082473637954
R${}^{2}$	0.9009540457897911

## Data Availability

Not applicable.

## Abbreviations

The following abbreviations are used in this manuscript:

ACF	Autocorrelation function
ARMA	Autoregressive moving average
BLE	Bluetooth low energy
FPE	Final prediction error
GPS	Global positioning system
IoT	Internet of Things
MAE	Mean absolute error
MSE	Mean squared error
PACF	Partial autocorrelation function
R2	Coefficient of determination or R-squared
RMSE	Root mean squared error
RSSI	Received signal strength indicator
SLAM	Simultaneous localization and mapping
